# Investigation of the Microstructure and Mechanical Properties of Copper-Graphite Composites Reinforced with Single-Crystal α-Al_2_O_3_ Fibres by Hot Isostatic Pressing

**DOI:** 10.3390/ma11060982

**Published:** 2018-06-11

**Authors:** Guihang Zhang, Xiaosong Jiang, ChangJun Qiao, Zhenyi Shao, Degui Zhu, Minhao Zhu, Victor Valcarcel

**Affiliations:** 1 School of Materials Science and Engineering, Southwest Jiaotong University, Chengdu 610031, Sichuan, China; guihangzhang@yeah.net (G.Z.); zysao_10227@163.com (Z.S.); dgzhu@home.swjtu.edu.cn (D.Z.); zhuminhao@swjtu.edu.cn (M.Z.); 2 Tsinghua Innovation Center in Dongguan, Advanced Composite Materials Research Department, Dongguan 523808, Guangdong, China; qiaocj@tsinghua-dg.org (C.Q.); info@neoker.org (V.V.)

**Keywords:** single-crystal α-Al_2_O_3_ fibres, copper-graphite, hot isostatic pressing, microstructure, strengthening mechanism

## Abstract

Single-crystal α-Al_2_O_3_ fibres can be utilized as a novel reinforcement in high-temperature composites owing to their high elastic modulus, chemical and thermal stability. Unlike non-oxide fibres and polycrystalline alumina fibres, high-temperature oxidation and polycrystalline particles boundary growth will not occur for single-crystal α-Al_2_O_3_ fibres. In this work, single-crystal α-Al_2_O_3_ whiskers and Al_2_O_3_ particles synergistic reinforced copper-graphite composites were fabricated by mechanical alloying and hot isostatic pressing techniques. The phase compositions, microstructures, and fracture morphologies of the composites were investigated using X-ray diffraction, a scanning electron microscope equipped with an X-ray energy-dispersive spectrometer (EDS), an electron probe microscopic analysis equipped with wavelength-dispersive spectrometer, and a transmission electron microscope equipped with EDS. The mechanical properties have been measured by a micro-hardness tester and electronic universal testing machine. The results show that the reinforcements were unevenly distributed in the matrix with the increase of their content and there were some micro-cracks located at the interface between the reinforcement and the matrix. With the increase of the Al_2_O_3_ whisker content, the compressive strength of the composites first increased and then decreased, while the hardness decreased. The fracture and strengthening mechanisms of the composite materials were explored on the basis of the structure and composition of the composites through the formation and function of the interface. The main strengthening mechanism in the composites was fine grain strengthening and solid solution strengthening. The fracture type of the composites was brittle fracture.

## 1. Introduction

Copper matrix composites not only have high strength, good electrical conductivity, and thermal conductivity, but also show high wear resistance and a low thermal expansion coefficient [1,2,3]. However, with the rapid development of aerospace and electronic power industries, traditional copper matrix composites can no longer satisfy the high requirements for the above-mentioned properties, and it is therefore highly important and essential to design and study copper matrix composites with better mechanical properties. Carbon nanotubes (CNTs) and graphene nano-sheets are widely used in copper matrix composites as reinforcement materials owing to their unique structure and excellent properties [4,5], leading to the greatly improved strength and the hardness of these composites. In addition to these materials, alumina (Al_2_O_3_) is one of the most important ceramic reinforcements that has relatively high melting (2054 °C) and boiling points (2980 °C), ultrahigh thermal stability, and mechanical strength. Addition of fine Al_2_O_3_ particles into the copper matrix can not only improve the hardness of this material, but also reduces the grain growth rate even at the temperatures approaching the melting point of the copper matrix [6,7]. Therefore, these kinds of composites are widely utilized in electronics, automobile, and aerospace fields as electrical contact materials, rocket thrusters, and components in aircraft engines, as well as in other applications [8,9]. By controlling the amount, size, and distribution of the reinforcing particles, it is possible to obtain a wide range of properties of the composites for different applications. With a length and diameter of 200 μm and 3.5 μm, respectively, alumina short fibres exhibit excellent properties, such as elastic modulus as high as 300 GPa, strength of 1000 MPa, and hardness reaching as high as 700 HV [10], making them excellent reinforcement materials and leading to their use in some aluminium and magnesium matrix composites [11]. Alumina whiskers also have excellent properties and composites fabricated using these reinforcement materials can gain higher properties. In spite of the introduction of the preparation methods of alumina whiskers by Valcarcel et al. [12] and Victor et al. [13], to date, these kinds of advanced materials have not been widely used as reinforcement materials.

Various powder metallurgy routes are available for the fabrication of the copper matrix composites reinforced with Al_2_O_3_ particles, including mechanical milling of Cu with alumina [14,15,16], internal oxidation of Cu-Al alloy powders followed by reduction [17,18,19], as well as the use of an in situ thermo-chemical technique [20,21,22,23]. In addition, some extensive methods such as equal channel extrusion and the rolling method can also be used for the fabrication of these composites [24,25]. Jena et al. prepared a copper-alumina composite by hydrogen (*P*(H_2_) = 10.1325 kPa) reduction of a homogeneous mixture of finely divided CuO and Al_2_O_3_. It was found that Al_2_O_3_ particles have a nanometre size and are uniformly distributed in the copper matrix. An appreciable mount of a third phase was found, which was suggested to be CuAlO_2_ [26]. Ramos et al. synthesized Cu-Ni/Al_2_O_3_ nanocomposites by a chemical-based synthesis and finally by consolidation into pellets, and showed that the majority of Al_2_O_3_ is formed at the newly generated grain boundaries of the consolidated pellet while a small fraction of Al_2_O_3_ is found in the metallic Cu-Ni matrix, yielding an increase in the hardness [27]. In recent years, the size of Al_2_O_3_-reinforced phase particles in copper-based composites was generally found to be on the nanoscale [28,29,30]. The strengthening mechanism of Al_2_O_3_-reinforced copper matrix composites is the typical diffusion strengthening [31,32]. Additionally, solid solution and precipitation strengthening are two very powerful approaches for improving the mechanical strength of Cu-based alloys [33]. According to recent studies, it is necessary and sufficient to add appropriate alloying elements such as Cr, Ag, Nb, and Zr into the copper matrix for which the solid solubility decreases with the lowering of the temperature [34,35,36,37]. However, these composites are not useful for high temperatures due to effects of recrystallization, particle coarsening and decomposition of the supersaturated solid solution [38,39]. Currently, two main problems hinder the further development of Al_2_O_3_-enhanced copper matrix composites: the first problem is the interfacial bonding caused by the wettability between particles and matrix [40], and the second problem is the tendency of nanometre-sized alumina particles to agglomerate [41].

In reviewing the literature, there are few papers on alumina whisker-reinforced copper matrix composites. Hence, single-crystal α-Al_2_O_3_ whisker and Al_2_O_3_ particles synergistic reinforced copper-graphite composites are researched in this study, which are synthesized by mechanical alloying and hot isostatic pressing (HIP) techniques. At the same time, various alloying elements were added to the composites, aiming to achieve multiple strengthening effects and improve the properties of composites. The present study was designed to determine the effect of whisker content on mechanical properties of the composites. Based on the mechanical properties and microstructure of the composites, the strengthening and fracture mechanisms were analysed.

## 2. Materials and Methods

**Figure 1:**
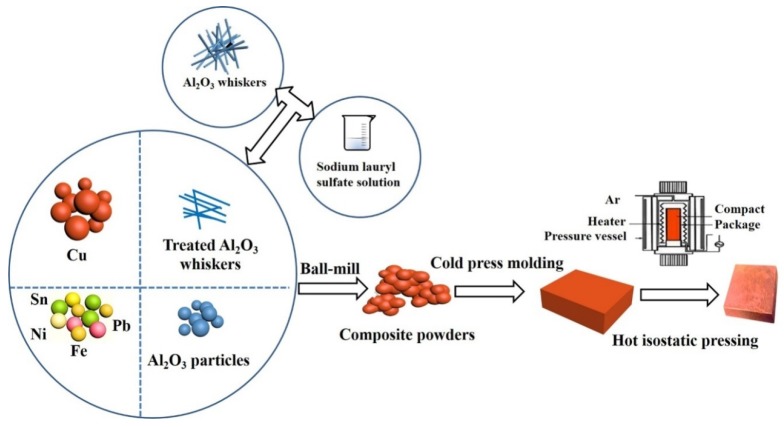
Schematic diagram of the procedures used to fabricate the copper matrix composites.

Al_2_O_3_ whiskers (purity > 99.7%) were supplied by Tsinghua Innovation Centrein Dongguan, Advanced Composite Materials Research Department (Dongguan, China). Al_2_O_3_ particles were obtained from Shanghai Jiahe Trading Co., Ltd. (Shanghai, China). Graphite was received from Handan Juxing Carbon Co., Ltd. (Handan, China). In order to improve the dispersion properties, Al_2_O_3_ whiskers were treated with a sodium dodecyl sulphate solution, followed by standing, filtration, and drying. The purity of raw materials used in this study was greater than 99.5%, and the specific particle sizes and density are shown in Table 1. In this experiment, the amount of Al_2_O_3_ whiskers and particles was changed, while keeping the other components the same. The total amount of alumina was 3 wt %, and the contents of Al_2_O_3_ whiskers were 0 wt %, 0.5 wt %, 1 wt %, and 1.5 wt %. The specific contents of graphite, Ni, Fe, Sn, Pb, ZrO_2_, La, and Cu are 7 wt %, 5 wt %, 5 wt %, 6 wt %, 4 wt %, 1.9 wt %, 0.1 wt % and 68 wt %, respectively. Copper matrix composites were designed based on the concept of a multi-component alloy system synergistic reinforced with alumina particles and whiskers, and prepared by powder metallurgy method of hot isostatic pressing which included mixing, compacting, and sintering. The preparation process of the composites is shown in Figure 1.

The mechanical properties and microscopic morphology of the composites were tested and analysed. The phase of composite powders and composites was analysed by X-ray diffraction (XRD, X-Pert PRO-MPD, PANalytical B.V., Almelo, The Netherlands) with a copper target. The composite microstructures and fracture morphologies were observed by a scanning electron microscope (SEM, JEOL JSM-7001F at 15 kV, JEOL Ltd., Tokyo, Japan) equipped with an X-ray energy-dispersive spectrometer (EDS). An EDS was used to observe the distribution of the elements in the detection area, and the distribution of the elements in the material was also observed by electron probe microscopic analysis (EPMA, JEOL JXA-8530F field-emission hyperprobe at 15 kV, JEOL Ltd., Tokyo, Japan) for elemental mapping using a wavelength-dispersive spectrometer (WDS, JEOL Ltd., Tokyo, Japan). The composite microstructure, particle size, and morphology were observed using a transmission electron microscope (TEM, FEI Tecnai F20ST, Hillsboro, OR, USA) equipped with EDS. A micro-hardness tester (HXD-1000TM-LCD, Shanghai Optical Instrument, Shanghai, China) was used to measure the micro-hardness of the sintered composites, the load was 1000 gf, and the holding time was 15 s. The compressive strength of the sample was tested using an electronic universal testing machine (WDW-3100, Guangzhou Precision Instrument Co., Ltd., Guangzhou, China).

## 3. Results and Discussion

### 3.1. Results of the Composites PowderAnalysis

**Figure 2:**
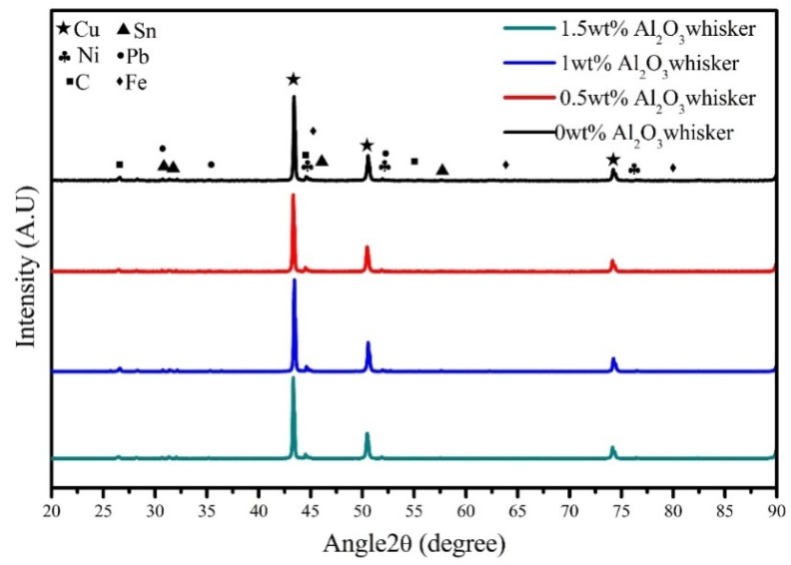
X-ray diffraction (XRD) patterns of powder materials with different content of alumina whiskers.

Figure 2 shows the XRD patterns of the powder materials with varying contents of alumina whiskers. The diffraction peaks at 2*θ* = 43.405°, 50.528°, and 74.211° are indexed to Cu, corresponding to the (111), (200), and (220) crystal planes. The peaks of Ni, Fe, C, Sn, and Pb are weak and are not apparent, whereas the diffraction peaks of Al_2_O_3_ whiskers and particles, ZrO_2_ particles, and La are not detected. This is attributed to their low content, and similar results have been reported in other studies [14,22]. Meanwhile, no CuO or oxide of other alloying elements was detected in the XRD pattern, indicating that no oxidation occurred during the milling process. Choi et al. [42] examined the grinding behaviour of copper particles in the preparation of CNT-reinforced copper matrix composites by the high-energy ball milling method. X-ray diffraction analysis indicated that the crystal structure was not changed during the milling process, and the raw material exists in its own preferred phase, which was consistent with the experimental results.

**Figure 3:**
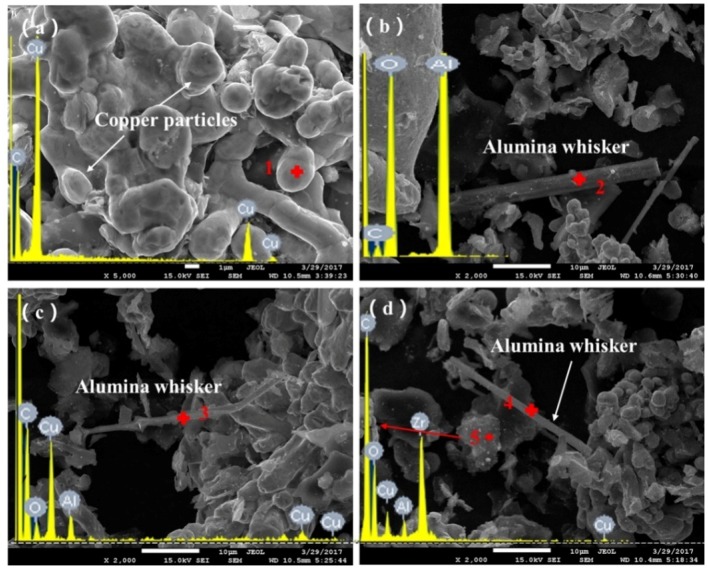
Scanning electron microscopy (SEM) micrographs of powder materials with different contents of alumina whiskers: (**a**) 0 wt %; (**b**) 0.5 wt %; (**c**) 1 wt %; (**d**) 1.5 wt %.

Figure 3 presents the SEM micrographs of powder materials with varying contents of alumina whiskers and the corresponding point of energy spectrum results. It can be seen from the figure that Cu particles appeared to be deformed. The particles’ size was approximately 1 μm, which is considerably smaller than that for the raw materials. The particles were ellipse to rod-shaped and were connected to each other. These results indicate that the compound powders had undergone repeated deformation, cold welding, as well as breaking and a series of physical and chemical processes during milling [43]. The powder particles exhibited significant deformation and exerted a cold welding effect. Several white particles were uniformly dispersed on the surface of the copper particles. Figure 3b,d show fibre-like materials consisting of alumina whiskers. As shown in Figure 3d, the white particles on the surface of copper particles represent the mixtures of Al_2_O_3_ and ZrO_2_ particles with the size of less than 1 μm. Compared to the size of the raw material particles, the sizes of the Al_2_O_3_ and ZrO_2_ particles were markedly reduced after ball milling. These results generally suggest that mixing between copper particles and reinforcements was uniform and the ball milling exerted a positive effect.

### 3.2. Microscopic Morphology Analysis of the Composites

**Figure 4:**
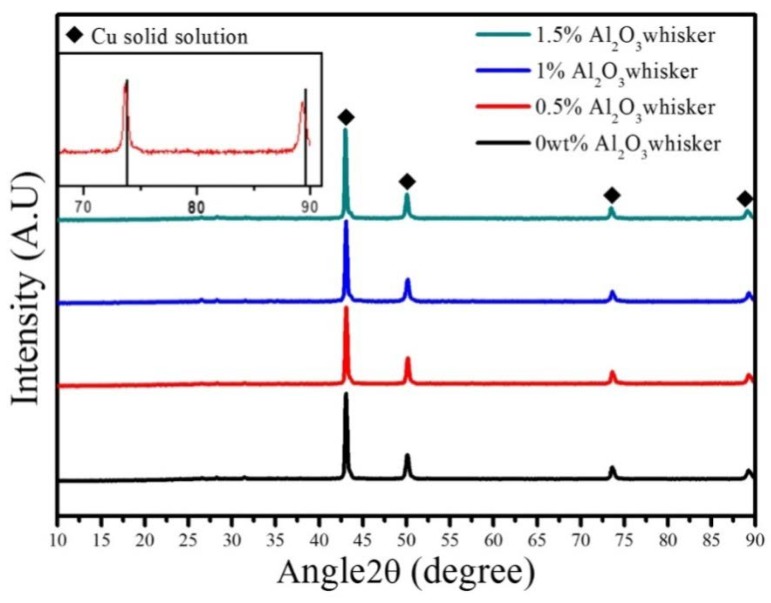
XRD patterns of the sintered composites with different contents of alumina whiskers. Comparison with copper standard card (70-3038) shown in the upper left panel.

Figure 4 shows the XRD patterns of sintered composites with varying contents of alumina whiskers. Only the Cu peaks were detected in the XRD patterns of the samples. The Ni, Fe, C, Sn, and Pb peaks were found to be virtually disappeared in comparison with results in presented in Figure 2. Additionally, the Cu peak shifted to the left. Compared to the XRD standard card (70-3038), the copper peak was clearly shifted to the left, as shown in the upper left panel of Figure 4. The Cu peaks of the (220) and (311) facets from 73.765° and 89.499° shifted to 73.577° and 89.245°, respectively. This indicates that an alloying element entered the copper crystal during the sintering process, causing the Cu lattice to be distorted. This effect increased the lattice constant and shifted the peaks to the left. Simultaneously, the weak peaks of alloying elements, such as Ni and Fe, disappeared. This effect was also observed by Zúberová et al. [24] in the study of copper alloys reinforced with nano-Al_2_O_3_ particles, which were prepared by mechanical alloying and equal channel angular pressing. After the alloying of Cu-4.5Cr and Cu-4.5Cr-3Ag for 25 h, the peak of the alloying element Cr(110) disappeared in the XRD pattern. It was considered that all Ag elements and most of the Cr elements in the two powder samples were solid-dissolved into the Cu crystals to form a single-crystal copper alloy phase, as was also reported in other studies [27,37].

**Figure 5:**
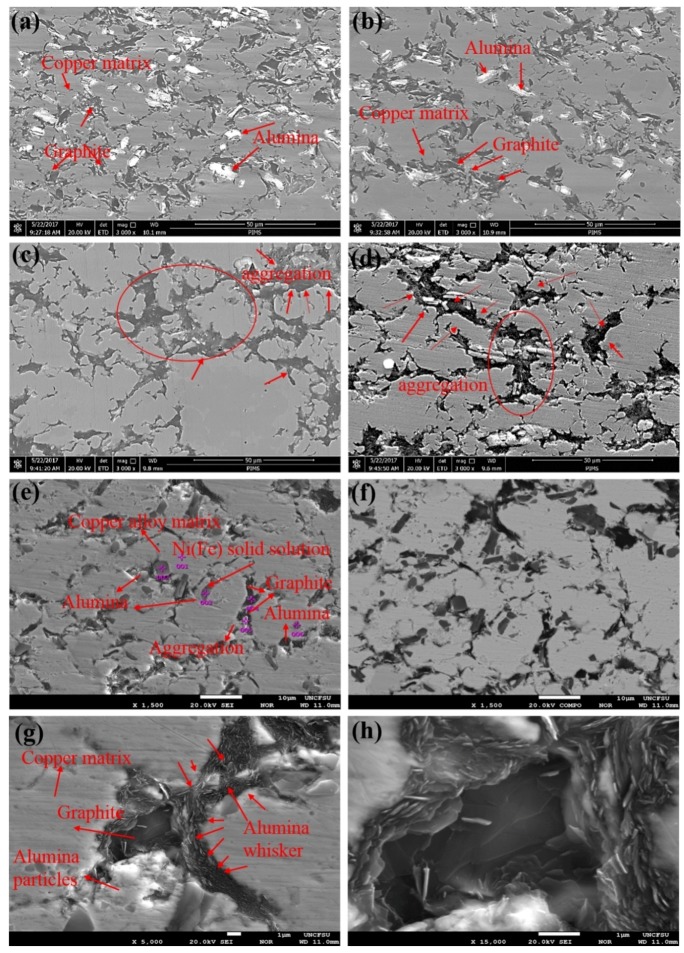
SEM micrographs of sintered composites with different contents of alumina whiskers: (**a**) 0 wt %; (**b**) 0.5 wt %; (**c**) 1 wt %; (**d**) 1.5 wt %; (**e**) SEM micrograph of sintered composites with 0.5 wt % alumina whiskers obtained by electron microprobe microanalysis; (**f**) corresponding backscattered elctrons(BSE) micrograph of (**e**); (**g**) SEM micrographs of sintered composites with 1 wt % alumina whiskers at 15,000×; (**h**) corresponding SEM micrograph of (**g**) at 25,000×.

To observe the distribution of the strengthening phase and various alloying elements in the matrix, the SEM images of copper matrix composites reinforced with different content of Al_2_O_3_ whiskers were obtained by scanning electron microscopy and electron probe microanalysis. Surface scanning results were also collected. The scanned images more clearly present the distribution of each phase, and reflect the bonding between the reinforcement and the Cu matrix. Figure 5 shows the micrographs of the sintered composites reinforced with different contents of alumina whiskers. It can be seen from Figure 5a,d that the samples are composed of three different phases, including the uniformly distributed white phase, the stripe-like distribution of the black phase, and most of the grey phase. Regarding the composition of the composite, the white phase consists of Al_2_O_3_ particles with meshes of 200 meshes. Owing to the continuous collision and deformation between the powders during ball milling, the particle size becomes refined, with 10 μm as the average size of the particles as shown in Figure 5a,b. Thus, the mechanical properties of the composites can be improved [44]. The black phase is graphite, which exhibited a homogeneous distribution and did not agglomerate. The bonding between graphite and the matrix was quite strong; most of the grey objects were copper substrates. The amounts of alumina particles in Figure 5c,d were clearly reduced. With an increase in alumina whiskers, the amount of alumina particles was reduced. Simultaneously, most of the alumina particles were distributed at the location of the graphite and were not easily observed. The graphite was largely aggregated to separate the copper matrix and the bonding between graphite and the matrix was not sufficiently strong, as shown in Figure 5c,d. Aggregation is volume defect in structural defect, and the presence of defect leads to a decrease of properties. For example, the compressive strength and micro-hardness of composites decrease, the strengthening effect of reinforcements fail to perform. When the material was used in practical applications, micro-cracks started to nucleate at the bonding site between graphite and the copper matrix, and then continued to expand, finally resulting in the failure of the material. To elucidate the presence and distribution of the Al_2_O_3_ particles and Al_2_O_3_ whiskers in sintered composites, the microstructures of the composites are characterized by EPMA. Secondary electrons and back scattered electron scans of the copper matrix composites are shown in Figure 5e,f, respectively, which were reinforced with 0.5 wt % alumina whiskers. As seen from Figure 5e,f, the composite consists of three parts, including the grey matrix, the darker grey structure, and the black cluster. This observation, combined with the results listed in Table 2 suggests that the microstructure of the grey matrix consists of Cu copper alloy. As can be inferred from the energy spectrum at point 1 in Table 2, the Cu alloy is mainly composed of Cu, Sn, Fe, and Ni. This indicates that the composite matrix is indeed a solid solution, consistent with the XRD results presented in Figure 4. The energy spectrum at point 3 in Table 2 confirms that the dark grey microstructure is Ni (Fe) solid solution, which is consistent with the results of the previously obtained energy spectrum. In Figure 5e,f, the black cluster consists of Al_2_O_3_ particles, graphite, and their clusters. Combining the point scanning results of several special locations in Table 2, it is possible to distinguish the Al_2_O_3_ phase and graphite may be observed as well; both exhibit a uniform distribution in the matrix. However, different phases of the enhancement phases are found in similar locations, enabling the formation of a weak interface or the existence of pores and other defects. The enhanced body cannot effectively play the role of dispersion enhancement. By contrast, the performance of the composite materials is reduced. Dash et al. evaluated the process and progress of sintering behaviour of Cu-Al_2_O_3_ composites [45]. The results also show that porosity and interfacial de-bonding influence the mechanical properties of the composites to a certain extent, which can reduce the bearing capacity. Composites basically have a homogeneous composition with few clusters that have the size of 5–10 μm. Figure 5g,h shows the SEM micrographs of sintered composites reinforced with 1.0 wt % alumina whiskers at a high degree of magnification. As shown in the figure, graphite is found in clusters in the composite, and numerous clusters of fibrous Al_2_O_3_ whiskers are distributed around the graphite clusters. This result indicates that the reinforcement in the matrix has an uneven distribution, and the interface between the reinforcement and the matrix is not good and contains the defects in the composite material.

**Figure 6:**
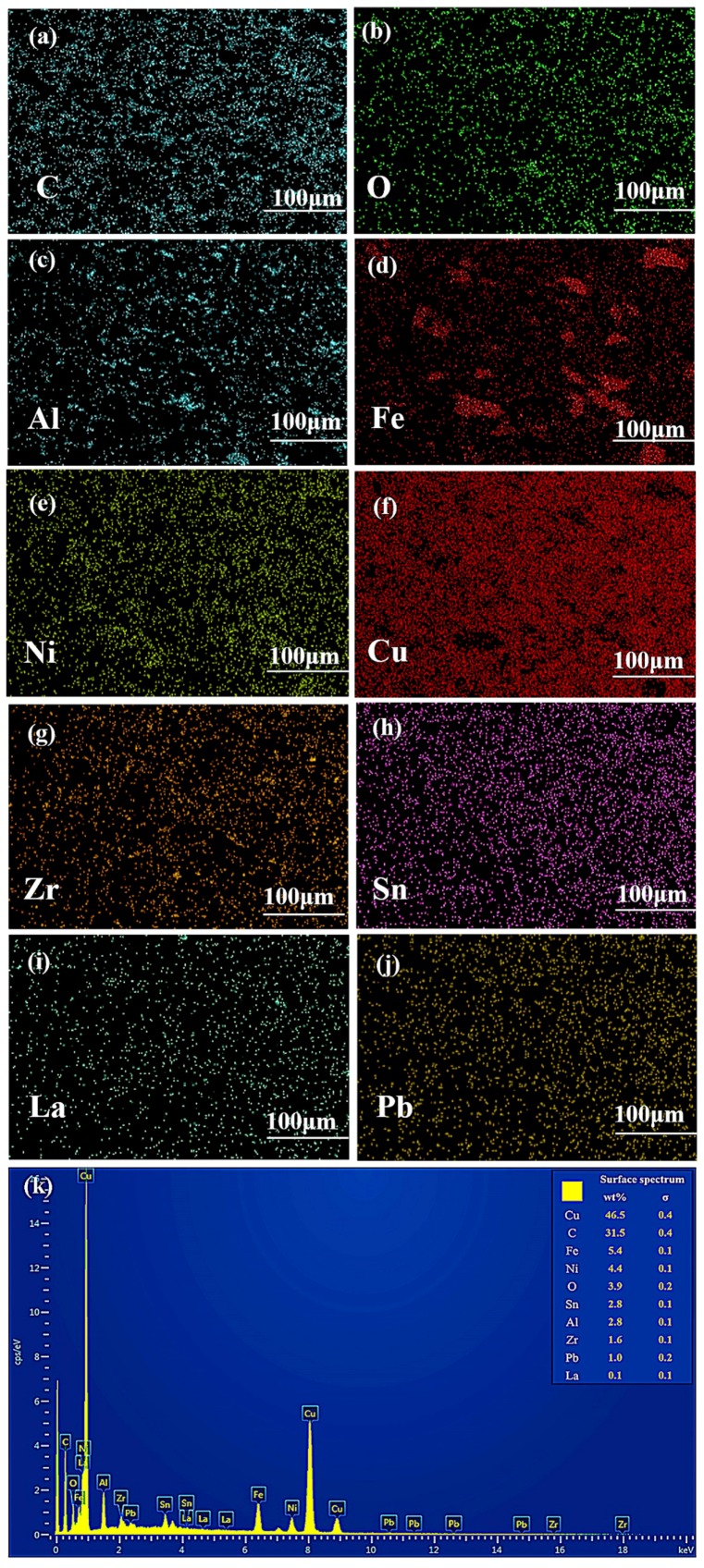
Corresponding EDS elemental mappings of sintered composites with 0.5 wt % alumina whiskers: (**a**) C; (**b**) O; (**c**) Al; (**d**) Fe; (**e**) Ni; (**f**) Cu; (**g**) Zr; (**h**) Sn; (**i**) La; (**j**) Pb; (**k**) surface spectrum.

Figure 6 shows the corresponding EDS elemental mappings of sintered composites reinforced with 0.5 wt % alumina whiskers. It is obvious from the figures that C, Ni, O, Cu, La, Sn, Zr, and Pd elements are evenly distributed, as clearly shown in the figure. This result shows that alumina particles were uniformly dispersed in the copper matrix, which is consistent with the SEM results presented in Figure 5b. Simultaneously, other alloying elements were uniformly distributed throughout the area, and a solid solution could be formed between these elements and the copper matrix. Moreover, Fe elements in a small part of the region were significantly enriched, and the distribution of the Cu elements exhibited a complementary relationship. Ni elements also show an enrichment phenomenon, which suggests that Fe and Ni can form a solid solution. Zúberová et al. [24] observed the same phenomenon in the study of Cu alloys reinforced nano-Al_2_O_3_ particles by mechanical alloying and equal channel angular pressing. As inferred from the results of the line scan of the composites, Cr forms a transition layer or a reaction layer on the surface of uniformly dispersed Al_2_O_3_ particles. Cr diffuses from a solid solution of Cu at higher temperatures and enriches on the surface of Al_2_O_3_ particles.

**Figure 7:**
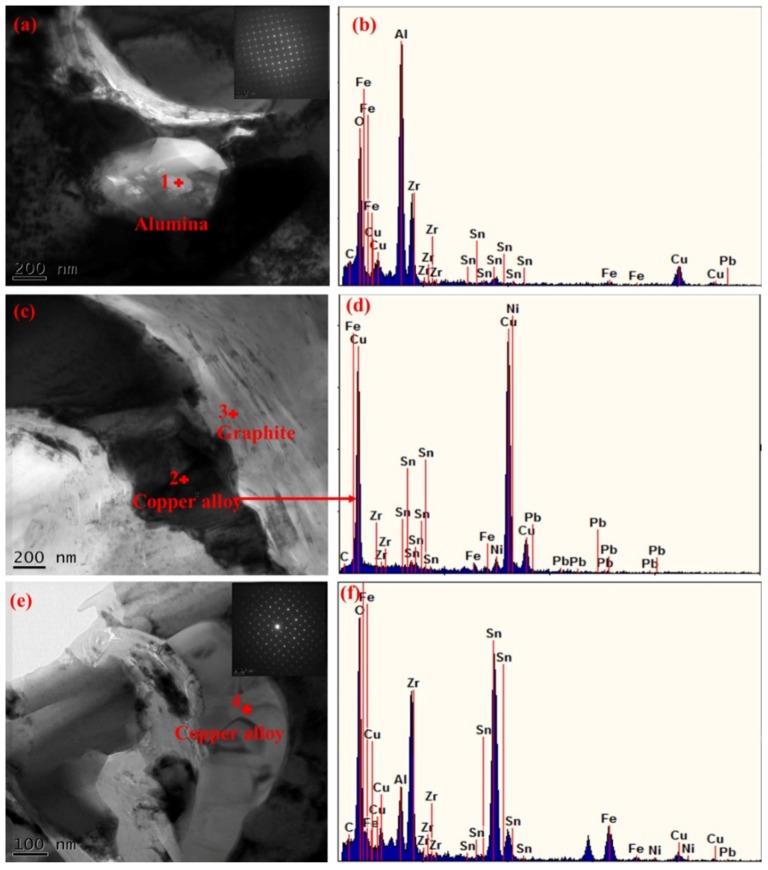
TEM images of sintered composites with 0.5 wt %alumina whiskers: (**a**) TEM bright field images, corresponding selected-area electron diffraction pattern obtained from point 1 shown in the upper right panel; (**b**) EDS analysis at point 1; (**c**) TEM bright field images; (**d**) EDS analysis at point 2; (**e**) TEM bright field images, corresponding selected-area electron diffraction pattern obtained from point 4 shown in the upper right panel; (**f**) EDS analysis at point 4.

Figure 7 presents the TEM images of sintered composites reinforced with 0.5 wt % alumina whiskers. Bright field microscopy images and corresponding energy spectrum results of point 1 are shown in Figure 7a,b, respectively. The image at the upper right corner of Figure 7a shows a selected-area electron diffraction pattern. The images, combined with the results of energy spectrum measurement lead to the conclusion that the electron diffraction pattern of the single crystals in Figure 7a were alloy phases formed by Sn, Fe, Cu, and other elements. The white particle at point 1 represents the Al_2_O_3_ particles. By further observation, it can be found that the size of Al_2_O_3_ particles was several hundred nanometres, and the particles were well-embedded in the copper matrix. According to the Orowan mechanism, the applied load is mainly borne by the Cu matrix, and the Al_2_O_3_ particles perform dispersion strengthening by hindering the movement of dislocations [46]. When a dislocation moves forward and meets a hard Al_2_O_3_ particle, it only moves around the particle, thus leaving a dislocation loop. This process requires energy; thus, the composites exhibit greater strength than the pure Cu [7]. Figure 7c presents the morphology of the composite in other regions, and Figure 7d shows the energy spectrum corresponding to point 2. These observations, combined with Figure 7d, indicate that the materials corresponding to points 2 and 3 are respectively copper alloy matrix and graphite. Some folds are present on the surface of graphite. In addition, no reaction layers were formed between the Cu matrix alloy and graphite, and they were combined by mechanical bonding. Kim et al. [47] examined the distribution of multi-layered graphite in the Cu matrix. High-resolution transmission electron microscopy results revealed that nano-sized graphite fragments adhered to the grain boundaries of the composites and formed a clear interface in the Cu matrix. No reaction occurred at the interface, and the bonding at the interface was also via the mechanical combination. The bright field microscopy images and the corresponding energy spectrum results of point 4 are shown in Figure 7e,f, respectively, and the image at the upper right corner of Figure 7e shows the selected area electron diffraction pattern. The images, combined with the energy spectrum results, lead to the conclusion that the electron diffraction pattern of single crystals in Figure 7e was alloy phases formed by Sn and other elements. However, this alloy phase was different from the alloy in Figure 7a because the crystal surface spacing of the diffraction spot is different. In summary, the composite material includes an Al_2_O_3_ particle reinforced phase, a Cu alloy matrix, and a very small amount of the single-crystal alloy phase in the composite material. Thus, the alloy phases and the copper alloy matrix have different compositions. The obtained results show that various solid solution structures were formed in the composites, confirming the results of the previous analysis of EPMA; meanwhile, the Al_2_O_3_ whiskers were too few to be observed.

### 3.3. Mechanical Properties of the Composites

**Figure 8:**
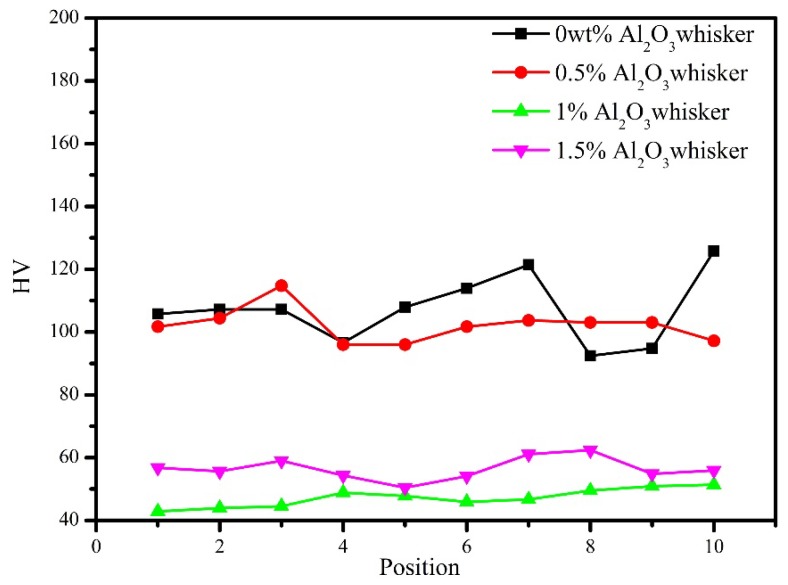
Micro-hardness of sintered composites with different content of alumina whiskers.

Figure 8 shows the micro-hardness of sintered composites reinforced with various contents of alumina whiskers. Micro-hardness is the type of press-in, and there is a certain relationship between the marked hardness value and the mechanical properties under static load, which can be used to obtain the approximate performance of other properties. The parallel hardness value of each specimen is expected to vary according to the principle of the micro-hardness measurement. The indentation size obtained by the micro-hardness measurement is small, and the organization varies frequently, resulting in a certain fluctuation in the parallel hardness value. To reduce the error, these four groups of samples were tested 10 times. The average data obtained from the four groups were reliable. When the total content of the Al_2_O_3_ whiskers was 0.5 wt %, the average hardness of the composites was reduced from 107.3 HV to 102.2 HV, and the content of the Al_2_O_3_ whiskers was therefore increased from 1 wt % to 1.5 wt %. In this case, the hardness values were slightly increased from 47 HV to 56 HV. The hardness of the composite material showed a generally decreasing trend. With an increase in the diameter, alumina whiskers were more likely to accumulate than the alumina particles, thereby reducing the bonding with the Cu matrix, as well as the hardness of the composites. With an increase in the alumina whisker content, the porosity of the composites increased and the density of the composites decreased, thereby reducing the hardness of the composite materials. Liu et al. [4] examined Ti_3_SiC_2_/C/MW-CNTs reinforced copper matrix composites and concluded that the hardness of composite materials decreases when the content of multi-walled carbon nanotubes (MW-CNTs) increased. The main reason is that MW-CNTs can easily agglomerate, promoting the formation of defects in the composite materials and destroying their integrity. Consequently, the hardness of the composite is reduced.

**Figure 9:**
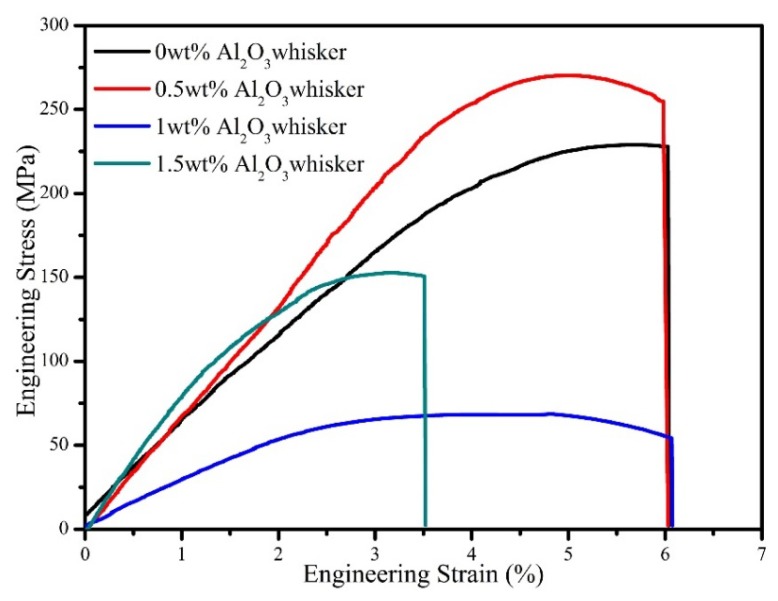
Compressive stress strain curve of sintered composites reinforced with different content of alumina whiskers.

Figure 9 shows the compressive stress-strain curves of sintered composites reinforced with different contents of alumina whiskers. As shown in the figure, the compressive strength of the composites first increased and then decreased with an increase in the alumina whisker content. Several studies have suggested that the overall strength of the composite materials can be improved by the introduction of ceramic particles into continuous fibre-reinforced aluminium matrix composites [48]. When the content of Al_2_O_3_ whiskers was increased to 0.5 wt %, the average compressive strength of the composites increased from 223 MPa to 247 MPa. This is because the presence of the particles can improve the stress transfer between the matrix and the reinforced fibre and render it more effective while reducing direct contact between the fibres and avoiding the occurrence of clusters and stress concentrations [10]. Together, the XRD and SEM results indicate that the composite powder produced a solid solution during the processing. Thus, a solution strengthening effect is produced. In addition, the composites show a grain-strengthening effect which is exerted by some alloying elements as well as the process of dynamic recovery and recrystallization. Stresses are produced because of the difference between the thermal expansion coefficients of the reinforcement and matrix materials; these stresses are partly eliminated at the sintering stage and are present to some degree in the form of dislocation initiation and multiplication, so that the composite exhibits a dislocation enhancement [49]. However, whereas the content of Al_2_O_3_ whiskers increased from 0.5 wt % to 1.5 wt %, the average compressive strength of the composites rapidly decreased to 76 MPa and slowly increased to 153 MPa. The SEM results presented in Figure 5g,h indicate the presences of numerous clusters of fibrous Al_2_O_3_ whiskers. The distribution of the reinforcement in the matrix was uneven, and the interface between the reinforcement and the matrix was not good. These parts are the location of defects in the composite material. When the sample is loaded, these positions often become a source of micro-cracks. With further loading, the cracks continued to expand, resulting in fracture.

Scherrer’s formula was used to calculate the size of crystallites as:

$$D=\frac{0.9\lambda }{\beta \cdot \cos\theta }$$

where D is the size of the crystallites, *λ* is the X-ray wavelength used, *β* is the broadening of the diffraction line measured as half of its maximum intensity, and *θ* is the corresponding angle. The results are listed in Table 3.

As shown in the table, the average size of the sintered composites in each crystal surface was smaller than the corresponding size of the composite powders. This difference results in the dynamic recovery and recrystallization of the samples after sintering. This phenomenon can be attributed to the dynamic recovery and recrystallization of the sintered sample. Composite powder was moulded under the pressure of 600 MPa. During the entire process, plastic deformation occurred after the composite powder was compressed. In other words, the work hardening phenomenon occurred. Therefore, after cold deformation, the deformation energy storage increased owing to the dislocation propagation as well as the increase in the vacancy and the presence of elastic stress, which provides the thermodynamic conditions for dynamic recovery recrystallization. The samples were subjected to hot isostatic pressing after cold deformation. When a certain temperature was reached and when the composite materials met the corresponding dynamic conditions, dynamic recovery and recrystallization occurred. Meanwhile, the presence of reinforcements such as Al_2_O_3_ particles, ZrO_2_ particles and alumina whiskers influenced the pinning [50], thus inhibiting grain growth to a certain extent [51]. Some alloying elements such as La, exerted an effect on the refining grain. In accordance with the principle of fine grain reinforcement, grain refinement can increase the mechanical properties of the composites [44]. The results show that compared to the four different contents of the composite materials, the average grain size of the composites increased slightly with a decrease in the Al_2_O_3_ particles, confirming that reinforcements give rise to pinning and suppression of grain growth.

The properties of metal matrix composites are influenced by many factors such as the size, shape and distribution of reinforcement. The interfacial bonding between the reinforcement and the matrix material arises owing to multiple factors. Nguyen et al. [44] demonstrated that increasing the amount of Ca leads to the refinement of the matrix grain size. In the current study, the particles and whiskers were used in two different shapes of reinforcement, and some alloying elements were added. This procedure could not only refine the grains and achieve fine-grained enhancement, but also forms a Cu solid solution to achieve solid solution strengthening. Therefore, the strengthening mechanism of the composite includes fine-grained enhancement, solid-solution strengthening and dislocation hardening.

### 3.4. The Compression Fracture Analysis of the Composites

**Figure 10:**
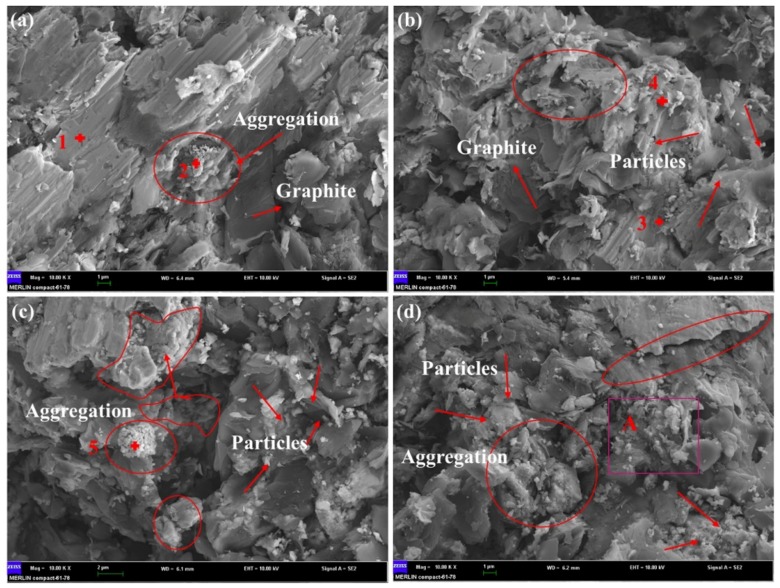
SEM micrographs of fracture surfaces of sintered composites with different content of alumina whiskers: (**a**) 0 wt %; (**b**) 0.5 wt %; (**c**) 1 wt %; (**d**) 1.5 wt %.

Figure 10 presents the SEM micrographs of the fracture surfaces of sintered composites reinforced with different contents of alumina whiskers. Table 4 shows the weight percentages of some special points on the fracture surfaces. As shown in the figure, the composite material exhibited a typical brittle fracture, with no apparent plastic deformation at the fracture site. No dimples, fibre and shear lips were observed. These observations, combined with the results in Table 4, indicate the occurrence of agglomeration phenomena in four types of samples, including large-scale graphite agglomeration and some particle aggregations. The aggregates in Figure 10c are aggregates of Al_2_O_3_ particles and ZrO_2_ particles. Generally, fewer defects were present in the clusters shown in Figure 10a,b, and the bonding between the reinforcement and the Cu alloy matrix was more compact. An enhancement effect can be exerted, showing an improvement in the mechanical properties of the composites. The defects observed in Figure 10c,d were larger, and were found in greater amounts than the defects in Figure 10a,b. Gaps existed between the reinforcement and the matrix, and interface bonding was not sufficiently strong, which is consistent with the results for the compressive strength of the composites.

**Figure 11:**
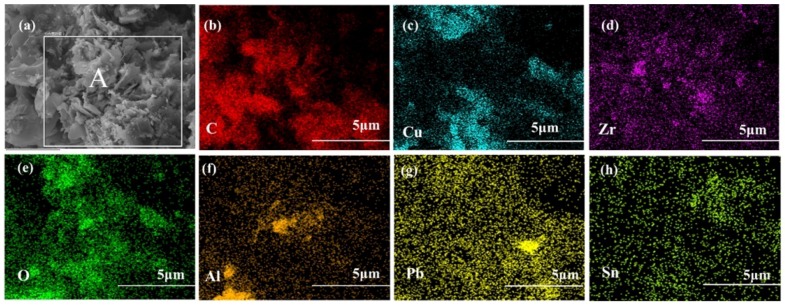
Corresponding EDS elemental mappings of micro-zone A in Figure 10d: (**a**) SEM of zone A; (**b**) C; (**c**) Cu; (**d**) Zr; (**e**) O; (**f**) Al; (**g**) Pb; (**h**) Sn.

Figure 11 presents the results of the surface scanning of area A in Figure 10d. The fracture consists of graphite, Al_2_O_3_ particles, ZrO_2_ particles, and Cu alloy matrix. These observations, combined with the morphology observations, indicate the presence of aggregates of Al_2_O_3_ particles, ZrO_2_ particles, and graphite. The combination of the composites did not lead to sufficient firmness resulting in the fracture. The location of these defects often resulted in stress concentration and other phenomena. When the sample was loaded, these positions often become a source of micro-cracks. With further loading, the cracks continued to expand, resulting in fracture. In other words, no enhancement occurred because of the presence of a defect, which destroyed the integrity of the matrix structure and influenced the performance of the composites.

The fracture mechanism of the composite material is the initiation of micro-crack induced by the stress concentration resulting from the internal defects of the material. The micro-cracks continue to expand under the external force and eventually lead to the occurrence of brittle fracture. Carreño-Morelli et al. [52] considered that the interface thermal stresses in metal matrix composites arise from the mismatch between the thermal expansion coefficients of the matrix and the reinforcement. These stresses can be relaxed by plastic deformation in the metal matrix, interface decohesion or crack initiation and propagation. The stress concentration between the interface and the matrix resulting from the mismatch and the stress concentration caused by the defects were mainly released by crack initiation and expansion.

## 4. Conclusions

(1) In this study, Al_2_O_3_-enriched copper matrix composites were successfully prepared by mechanical alloying and hot isostatic pressing. When the content of Al_2_O_3_ whiskers was in the range of 0–1.5 wt %, the hardness of the composites decreased with the increase of the Al_2_O_3_ whisker content, while the compressive strength of the composites first increased and then decreased with the increase of the Al_2_O_3_whisker content.
(2) The main strengthening mechanism in the composites was fine grain strengthening and solid solution strengthening, and Orowan mechanism and dislocation strengthening were also present. It can be seen from the XRD results that the process of dynamic recovery and recrystallization occurred during the sintering process, and the presence of La and other alloying elements played the role of refining grain, and the composites also showed a fine grain strengthening effect. Combining the results of EPMA and TEM, it was known that the composites showed a solid solution phenomenon in the sintering process, therefore, a solution strengthening effect was present. Additionally, Al_2_O_3_ particles and other reinforcements embedded in the copper matrix could show the strengthening effect of dislocation and Orowan mechanism.
(3) The fracture type of the composites was brittle fracture. From results of SEM and EPMA, it can be seen that holes, clusters of the reinforcements, and weak interface bonding were present in the composite, naturally causing stress concentration at the positions of these defects. When the sample was loaded, these positions often become an area of micro-crack source, and with further loadings, cracks continued to expand, resulting in fracture.

## Figures and Tables

**Table 1:** Properties of raw material powders.

Original Materials	Graphite	Alumina Whisker	Al_2_O_3_	Ni	Fe	Sn	Pb	ZrO_2_	La	Cu
Density (g/cm^3^)	2.2	3.99	3.9	8.91	7.86	7.28	11.34	5.85	6.7	8.9
Mesh number	200	Diameter, μm: 0.1–4 Length, μm: 5–30	200	250	250	250	250	200	-	250

**Table 2:** Summary of Energy-dispersive spectrometer(EDS) analysis conducted at selected locations shown in Figure 5e.

Elements	C	O	Al	Fe	Ni	Cu	Sn
Point 1	0	0	0	2.32	4.62	87.56	5.5
Point 2	0	45.26	46.20	0	0	8.54	0
Point 3	0	35.33	32.12	0	1.56	28.71	2.28
Point 4	84.39	0	0	0	0	15.61	0
Point 5	53.07	10.96	9.50	3.57	2.11	18.98	1.82
Point 6	0	5.64	8.35	1.18	4.30	75.25	5.27

**Table 3:** Mean grain sizes of Cu composite powders and composite at different crystal planes.

Samples	Alumina Whiskers Content (wt %)	(111) Crystal Plane	(200) Crystal Plane	(220) Crystal Plane
Composite powders	0	576.5	351.5	357.5
	0.5	584	393	358.5
	1	625.5	407	420.5
	1.5	611.5	385.5	435.5
Composites	0	316	217.5	238
	0.5	324	264	256.5
	1	339.5	223	239.5
	1.5	357	249.5	242.5

**Table 4:** Summary of EDS analysis conducted at selected locations shown in Figure 10.

Elements	C	O	Al	Fe	Ni	Cu	Zr	Sn	Pb
Point 1	67.29	0	1.10	0.84	1.57	28.73	0.90	0.57	0
Point 2	32.93	6.94	2.63	5.91	3.66	37.71	8.16	2.06	0
Point 3	61.99	5.00	2.12	0.92	1.36	25.02	0	1.28	2.30
Point 4	27.29	9.79	0.54	1.16	2.83	43.91	11.90	2.58	0
Point 5	34.23	19.10	9.30	0	0	7.22	30.15	0	0
